# Using an Accelerometer-Based Step Counter in Post-Stroke Patients: Validation of a Low-Cost Tool †

**DOI:** 10.3390/ijerph17093177

**Published:** 2020-05-02

**Authors:** Francesco Negrini, Giulio Gasperini, Eleonora Guanziroli, Jacopo Antonino Vitale, Giuseppe Banfi, Franco Molteni

**Affiliations:** 1 IRCCS Istituto Ortopedico Galeazzi, 20161 Milan, Italy; francesco.negrini@gmail.com (F.N.); banfi.giuseppe@fondazionesanraffaele.it (G.B.); 2 Ospedale Valduce, Clinica Villa Beretta, 23845 Costa Masnaga, Italy; gasperini.giulio@virgilio.it (G.G.); eleonora.guanziroli@gmail.com (E.G.); fmolteni@valduce.it (F.M.); 3 Vita-Salute San Raffaele University, 20132 Milan, Italy

**Keywords:** step counter, accelerometer, stroke, gait, rehabilitation

## Abstract

Monitoring the real-life mobility of stroke patients could be extremely useful for clinicians. Step counters are a widely accessible, portable, and cheap technology that can be used to monitor patients in different environments. The aim of this study was to validate a low-cost commercial tri-axial accelerometer-based step counter for stroke patients and to determine the best positioning of the step counter (wrists, ankles, and waist). Ten healthy subjects and 43 post-stroke patients were enrolled and performed four validated clinical tests (10 m, 50 m, and 6 min walking tests and timed up and go tests) while wearing five step counters in different positions while a trained operator counted the number of steps executed in each test manually. Data from step counters and those collected manually were compared using the intraclass coefficient correlation and mean average percentage error. The Bland–Altman plot was also used to describe agreement between the two quantitative measurements (step counter vs. manual counting). During walking tests in healthy subjects, the best reliability was found for lower limbs and waist placement (intraclass coefficient correlations (ICCs) from 0.46 to 0.99), and weak reliability was observed for upper limb placement in every test (ICCs from 0.06 to 0.38). On the contrary, in post-stroke patients, moderate reliability was found only for the lower limbs in the 6 min walking test (healthy ankle ICC: 0.69; pathological ankle ICC: 0.70). Furthermore, the Bland–Altman plot highlighted large average discrepancies between methods for the pathological group. However, while the step counter was not able to reliably determine steps for slow patients, when applied to the healthy ankle of patients who walked faster than 0.8 m/s, it counted steps with excellent precision, similar to that seen in the healthy subjects (ICCs from 0.36 to 0.99). These findings show that a low-cost accelerometer-based step counter could be useful for measuring mobility in select high-performance patients and could be used in clinical and real-world settings.

## 1. Introduction

One of the main and most common issues of post-stroke patients is reduced mobility [1] due to the severe impairment of gait [2]. Moreover, reduced walking speed, asymmetry in spatio-temporal gait parameters, lack of a caregiver, and other environmental factors [3] can influence community ambulation [4] and physical activity [5]. Therefore, the measurement of gait is central to the classification of patients’ gait function and to evaluate the efficacy of rehabilitation interventions in order to improve clinical practice.

Gait analysis, usually performed in a movement analysis laboratory, can quantify spatio-temporal, kinematic, and kinetic parameters after stroke and help to identify walking patterns. However, movement analysis laboratories require expensive equipment, technical support, and a dedicated room. These environments provide a complete instantaneous view of a patient’s condition (capacity) but do not provide information about day-to-day variability in performance in a real-world setting [6]. Many valid and reliable outcome measures for mobility and walking ability in post-stroke patients are available in the scientific literature [7], and they are often combined in order to gain a more complete picture of global functioning. Most of these tests (i.e., 10 and 50 m walking tests, timed up and go test) are feasible, easy to perform, and repeatable, but they are also bound to a controlled and artificial setting to obtain standardized conditions [7]. In fact, these tests measure patients’ actual capacity, but they do not allow for the investigation of real-life performance [8]. An ecologic measure that is able to really quantify what a patient can do in a real-world setting could provide useful information on patients’ conditions and social participation to clinicians [9]. Questionnaires on self-reported physical activity have been proposed, such as the International Physical Activity Questionnaire (IPAQ) [10], the General Practice Physical Activity Questionnaire [11], and the Nordic Physical Activity Questionnaire—short [12]. Nonetheless, such tools may not be considered to be objective measures, because they strongly depend on patients’ perceptions and reliability. An objective test that is able to follow up with patients in their daily lives without interfering but continuously collecting data could give clinicians further information of the utmost importance. Even simply the number of steps per day, if accurately gathered, could allow clinicians to tailor treatments according to specific patients’ needs, taking into account real-life variables. In fact, it has been suggested that a change in the number of steps performed per day seems to influence health outcomes, and the effect of this is currently under scrutiny by the scientific community [13]. 

Technologic advancement and improvement in step counter development could help clinicians to obtain new and more reliable data on real-life patients’ mobility [14]. Step counters are light, portable, cheap, and easy to use [15,16], and their diffusion is wide; almost every smart phone has a built-in tri-axial accelerometer/step counter and the capability to act as a step counter with the appropriate software [17]. Up to now, devices combining different sensors and technologies have been used for the continuous monitoring of exercise activity, assessing performance, and monitoring sport activities [18,19,20]. A 2014 study shows that there could be a significant difference between physical activity quantified using a questionnaire, such as IPAQ, and physical activity objectively measured using step counters [21]. The accuracy of the sensors used in wearable devices is one of the main issues in the current commercial market, and currently, accelerometers are able to produce reliable data on walking or running when applied on the waist, thighs, or ankles [22,23]. While a wide number of sensors are commonly implemented in non-invasive wearable devices for healthy people [24], research examining the application of wearables to measure gait outcomes in stroke is emerging.

Step counters can be used to detect post-stroke patients’ activity levels [25,26,27], assess gait and balance parameters [28,29], and guide the patients in performing exercises [30], as well as acting as a stimulus to increase in- and out-patient activity levels [14]. The literature shows that the monitoring of patients’ mobility in an inpatient setting can give information similar to advanced and time-consuming techniques, such as behavioral mapping, and it can be useful to collect the activity levels of hospitalized patients [31]. A lower level of activity during hospitalization can lead to a loss of muscle quantity and quality and can seriously hurt the rehabilitation process and patient’s health conditions [32,33,34]. A review on wearable sensors for the quantification of, and feedback on, lower extremity movement found that there may be substantial clinical benefits, with more work needed with respect to natural environments and longer monitoring periods [35]. A recent Cochrane review confirmed the interest of scientific literature in the use of accelerometers in stroke patients, investigating the actual role of increasing global activity on the condition of adult stroke survivors; however, this review concluded that there is not enough evidence to support the regular use of accelerometers in clinical practice [14]. 

In the literature, it is possible to find many examples of step counters with built in accelerometers based on different and specific activity tracker models in stroke populations [36,37,38]. Although this application of wearable technology is promising, a few potential problems require consideration in stroke patients. The accuracy of commercial step counters has been questioned in stroke patients because the algorithm used to detect steps and spatio-temporal parameters is based on a cyclical, linear, and healthy model. Strokes can modify gait in many different ways, depending both on the impairment and on the strategies used to overcome the impairment; generally, stroke patients tend to increase the use of their unaffected side to support and balance body weight, causing reduced speed and gait asymmetry [39]. This can lead to serious problems in step counting, especially if asymmetry is not considered in the positioning of accelerometers [38]. For a realistic implementation of wearable technology in the clinical setting, while a wide number of sensors are commonly implemented in non-invasive wearable devices [24], it is very important to limit the number of sensor devices used for step detection [40]. To minimize the time expenditure of clinicians, it is necessary to use the fewest number of devices to assess performance.

There are no clear standards on where to apply accelerometers or guidelines on the patients for which accelerometers could be a valid and reliable option for measuring ecological mobility (real-life mobility, measure in an everyday setting [41]) after stroke and concerning neurological conditions in general, with the presence of only preliminary studies [42]. 

Furthermore, sophisticated accelerometer-sensor technologies have not been widely adopted outside the research realm, in part because of the high cost and complex software [43]. Therefore, considering the relatively low cost of step counters, their diffusion could not only be limited to high-income countries, but they could also be used in low–middle-income countries (LMIC) [44]. In fact, it has been reported that poorer patients have very low levels of physical activity [45] and have the worst functional outcomes post-stroke, so rehabilitation strategies targeting underprivileged populations in LMIC are needed [46]. A widely available low-cost instrument could provide an answer to this need.

The aim of this study is to validate a low-cost commercially available tri-axial accelerometer-based step counter for stroke patients by comparing five different positions of the step counter (wrist and ankle on the unaffected and pathological side and waist).

## 2. Materials and Methods

### 2.1. Design of the Study

This was a cross-sectional observational study aiming to evaluate the difference between manually and instrumentally counted steps for every position (N = 5) of the step counter and for every functional test (N = 4) for both healthy (N = 10) and pathological subjects (N = 43). Before entering the study, the participants were fully informed about the study aims and procedures, and written informed consent was obtained before testing. All subjects gave their informed consent for inclusion before they participated in the study. The study was conducted in accordance with the Declaration of Helsinki, and the protocol was approved by the Ethics Committee of “Comitato Etico Interaziendale delle Province di Lecco, Como, Sondrio” on the 15th of December 2016, with the number 272/2016.

### 2.2. Setting

The study was conducted in the laboratory of Neurological Rehabilitation Department of Valduce Hospital, Costa Masnaga (Italy).

### 2.3. Device

A commercial tri-axial accelerometer-based step counter named Nakosite 3D Walking was used. It is based on a triaxial accelerometer that uses proprietary algorithms to count steps and to estimate the distance covered and calories consumed using anthropometric parameters. Specific information regarding the threshold for activity intensity was unavailable from the device manufacturer or support staff. The device weighs 28 g, and its measurements are 7.8 × 3.4 × 1.2 cm. This model has a built-in 30-day memory that could help to follow the patient over time. Its price was, at the time of the study, one of the lowest on the market. No validation study of this device in neurological patients was found in the scientific literature. All step counters were fixed tightly in their positions using a Velcro system. For every trial, a specialist doctor checked the stability of the step counters; this was necessary to obtain reliable data. Five step counters were positioned in five different body positions:

1. Left upper limb on the dorsal side of the wrist;
2. Right upper limb on the dorsal side of the wrist;
3. Left lower limb on the internal malleolus;
4. Right lower limb on the internal malleolus;
5. On the waist, medially, on a line connecting the anterior superior iliac spine (ASIS).

### 2.4. Population

Ten healthy adult subjects (18 years old or older) without any major concomitant neurological, orthopedic, or psychiatric pathologies that influence gait pattern were enrolled. The healthy subject group was composed of five male and five female subjects. The average age was 36.6 years (±11.4).

Forty-three in- and out-patients from a neurological rehabilitation department were enrolled in the study. The post-stroke group was composed of 43 patients, 27 males and 16 females. The average age was 61.3 (±14.95) years. Twenty patients had a left hemiparesis; 18 patients had a right hemiparesis. The median time from the stroke was 359 days (26 years max; 13 days min). Six patients had suffered a stroke less than 1 month before, 10 patients had suffered a stroke between one and six months prior to the study, and 27 patients had suffered a stroke more than six months before the study. A total of 23 patients were able to walk without walking aids, 13 patients needed a unilateral cane to walk, and 7 needed a walker. The average spontaneous walking speed was 0.75 m/s (±0.32).

### 2.5. Inclusion/Exclusion Criteria

Patients were evaluated by a referent specialist doctor in order to verify their compliance with the inclusion and exclusion criteria. The inclusion criteria were as follows: (1) hemiparetic post-stroke patients (ischemic or hemorrhagic, acute, subacute, or chronic) and (2) the ability to perform the timed up and go (TUG) test without the assistance of an operator (supervision was allowed). The exclusion criteria were as follows: (1) major concomitant neurological, orthopedic, or psychiatric pathologies that influence deambulatory pattern or (2) inability to complete the TUG test. 

### 2.6. Experimental Procedures

During the execution of each functional test, a trained operator manually counted the steps using a mechanical step counter; in this way, a comparison between steps counted manually (gold standard) [47,48,49] and those collected by the five step counters was possible. Therefore, while wearing five step counters, the healthy subjects and patients performed the following clinical tests:

1. 10 m walking test at a self-selected speed (10 m WT) [50];
2. 50 m walking test (50 m WT) [51];
3. 6 min walking test (6 min WT) [7];
4. Timed up and go test (TUG) [7].

The 10 and 50 m walking tests consist of a 10 or 50 m walk on plain terrain at a self-selected speed and can clinically measure the comfortable walking speed of patients. The 6 min walking test consists of measuring the distance that the patient is able to walk in 6 minutes, thus investigating the patient’s endurance. The TUG test consists of getting up from a chair, walking 3 m, changing direction, walking 3 m, and sitting again on a chair. This test focuses more on balance, posture transitions, and change of direction. These clinical tests evaluate the indispensable requirements for walking independently and effectively in everyday life [52].

The order of execution of the test was always the same with a five-minute pause in-between tests: 1. TUG, 2. 10 m WT, 3. 50 m WT, 4. 6 min WT. 

### 2.7. Data Stratification of Stroke Patients 

Stratification was applied ex-post to two variables: (1) patients’ walking speed measured from the 50 m walking test and (2) walking aids used during walking.

Post-stroke patients were divided into three groups depending on their self-selected walking speed measured during the 50 m walking test, according to the classification proposed by Perry et al. [53]. Group V1 included patients with a walking speed of slower than 0.4 m/s. Group V2 included patients with a walking speed between 0.4 and 0.8 m/s. Group V3 included patients with a walking speed of faster than 0.8 m/s. Based on this classification, gait velocity in stroke patients was stratified into three clinically functional ambulation classes: household ambulation (<0.4 m/s), limited community ambulation (0.4 to 0.8 m/s), and full community ambulation (>0.8 m/s). In the first group (V1 group), we decided to include patients with a walking speed of less than 0.58 m/s on the basis of literature data according to which a speed lower than 0.58 m/s could create trouble with step detection by step counters [54,55].

Patients were also stratified on the basis of walking aids used during walking: the first group (A1 group) included patients who were able to perform the tests without any walking aid, the second group (A2 group) included patients who needed a unilateral aid (e.g., walking stick, unilateral crutch), and the third group (A3 group) included patients who needed a bilateral aid (e.g., walker).

Furthermore, time from stroke, expressed in days, was evaluated, and the patients were classified into three groups: less than 30 days, between 31 and 180 days, more than 180 days for further analysis.

The demographic and clinical characteristics of the groups are described in Table 1.

### 2.8. Data Analysis

Descriptive statistics (mean ± SD) for the outcome measures were calculated. The mean absolute percentage error (MAPE), the absolute value of the mean of the difference between manually and instrumentally counted steps expressed as a percentage, and the intraclass correlation coefficient (ICC) with a 95% confidence interval (CI) were calculated for every position of the step counter and for every test for both healthy and pathological subjects. Reliability was defined as poor when the ICC < 0.5, moderate when the ICC was between 0.5 and 0.75, good when the ICC was between 0.75 and 0.9, and excellent when the ICC > 0.9 [56]. In addition, the Bland–Altman plot was utilized to evaluate the bias between the mean differences of the two measurement methods (manual counting vs. step counter) during the TUG test, for each of the five different step counter positionings in both healthy and pathological groups.

The normality of the distribution of the subjects’ age at baseline was checked using the D’Agostino Pearson test. One-way analysis of variance (ANOVA) tests followed by Tukey’s multiple comparisons were performed to verify the differences in terms of age among V1, V2 and V3 and among A1, A2, and A3. In addition, Chi-square tests were done to check possible differences in terms of sex, walking aids, and distribution of distance from the stroke among the three groups of self-selected walking speeds and between the three groups for use of walking aids. Furthermore, a one-way ANOVA followed by Tukey’s multiple comparisons, or the equivalent non-parametric Friedman’s test when needed, was performed to compare MAPE among healthy, pathological, and V3 group, separately for each step counter positioning and in each of the four clinical tests. The level of significance was set at *p* < 0.05. Statistical analysis was performed using GraphPad Prism version 8.00 (GraphPad Software, San Diego, CA, USA). 

## 3. Results

Table 1 shows the patients’ sex distribution, age, mean gait speed, and use of walking aids between V1, V2, and V3 and A1, A2, and A3. No statistical differences were found among the three groups V1, V2, and V3 in terms of age (*p* = 0.645) and time from stroke (*p* = 0.795), while there were statistically significant differences in terms of sex (*p* = 0.030) and use of walking aids (*p* = 0.001). In particular, group V2 had a higher percentage of females in comparison with the other two groups, and group V1 needed fewer walking aids than the other two groups.

No statistical differences were found among the three groups A1, A2, and A3 in terms of age (*p* = 0.092), sex (*p* = 0.834), and time from stroke (*p* = 0.123), while there was a statistically significant difference in terms of comfortable walking speed (*p* <0.001), where patients who did not use any walking aid (group A1) walked faster than those in the other two groups. 

### Functional Tests Results

Healthy subjects mean self-selected walking speed was 1.2 m/s. During walking tests in healthy subjects, the best reliability was found for lower limbs and waist placement (ICCs from 0.46 to 0.99), while weak reliability was found for upper limb placement in every test (ICCs from 0.06 to 0.36) (Table 2). The TUG test showed weak step counter reliability for both upper and lower limbs placement but not for waist placement (95% CI—0.13–0.85).

In stroke patients, moderate reliability was found only for the lower limbs in the 6 min WT (healthy ankle ICC 0.69 95% CI 0.50–0.82; pathological ankle ICC 0.70 95% CI 0.51–0.83) (Table 3). For groups V1 and V2, all tests considered did not show any good or excellent correlations, while for group V3, excellent correlations were shown for all walking tests (10 m WT, 50 m WT, 6 min WT) between step counters positioned at the ankles and steps counted manually, except for the step counter placed on the affected ankle, which showed poor reliability in the 10 m WT (ICC 0.46 (95% CI—0.19–0.83) (Table 4).

**Figure 1:**
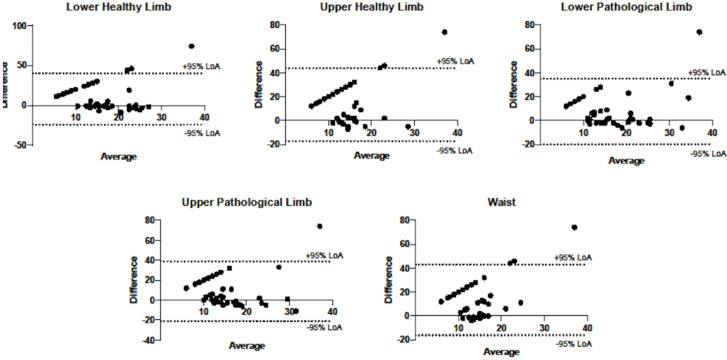
Bland–Altman plots for difference vs. average of the two measurements methods (step counter and manual counting) during timed up and go test (TUG) for the five different step counter positionings for the pathological group (*n* = 43). Dashed lines represent +95% (upper line) and −95% (lower line) of the limits of agreements. Legend: LoA, Limits of Agreement.

The Bland–Altman plot highlighted low mean values of bias (average of differences between step counter and manual counting) for the healthy group during the TUG test, specifically: right lower limb = 0.6; right upper limb = 2.5; left lower limb = 1.9; left upper limb = 3.0; waist = 1.7. On the contrary, the entire group of post-stroke patients registered high bias values and big average discrepancies between methods, in detail: right lower limb = 7.9; right upper limb = 13.6; left lower limb = 7.8; left upper limb = 9.1; waist = 13.5. Figure 1 shows the Bland–Altman plots, for the five different step counter positionings for the pathological group during TUG.

**Figure 2:**
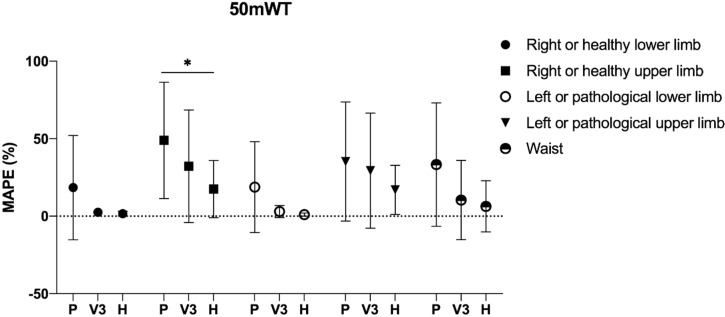
Comparison of the mean absolute percentage error in the 50 m walking test (50 m WT) for the healthy group (*n* = 10), pathological group (*n* = 43), and group V3 (*n* = 17). Legend: P: pathological group; H: healthy group; *: *p* < 0.05.

**Figure 3:**
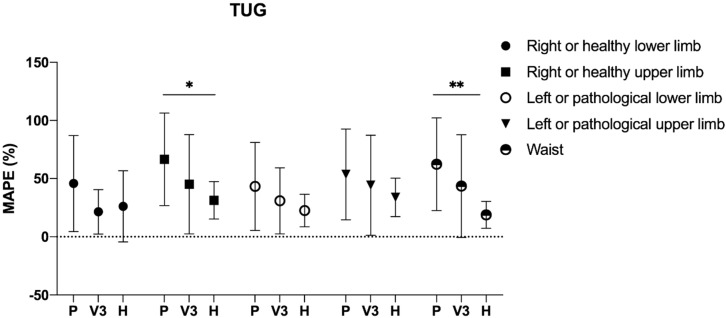
Comparison of the mean absolute percentage error in the timed up and go test for the healthy group (*n* = 10), pathological group (*n* = 43), and group V3 (*n* = 17). Legend: P: pathological group; H: healthy group; *: *p* < 0.05; **: *p* < 0.001.

The comparison of the MAPE, separately for each clinical test, among the healthy group, pathological group, and V3 highlighted significant differences in some of the five step counter positionings. In detail, one-way ANOVA was significant in 50 m WT for right or healthy upper limb (*p* = 0.027), left or pathological lower limb (*p* = 0.022), and waist (*p* = 0.021) with a post-hoc difference between the healthy and the pathological group only for the right or healthy upper limb (*p* = 0.035) (Figure 2); in addition, significant differences were detected in the TUG test for right or healthy upper limb (*p* = 0.015), right or healthy lower limb (*p* = 0.039), and waist (*p* = 0.004) with a post-hoc difference between the healthy and the pathological group only for the right or healthy upper limb (*p* = 0.027) and waist (*p* = 0.005) (Figure 3).

## 4. Discussion

Step counters have been identified as a useful device for measuring the mobility of post-stroke patients in an ecological way [15]. Even though some authors have demonstrated the possibility of quantifying the level of activity and gait pattern of healthy subjects with a single wearable sensor, this functionality has yet to be confirmed in stroke patients [25]. The quantification of stroke mobility is a new and very important concept because changes in steps per day seem to strongly influence health and global functioning outcomes [13]. 

Activity tracking devices that provide an objective measure of step count are commercially available tools; they have gained increasing popularity among people with different neurological conditions due to their user-friendly characteristics that make them easily usable outside the clinical setting. However, some concerns remain regarding the accuracy and real cost-effectiveness of the devices. For this reason, this study conducted an accurate validation assessment of a low-cost tri-axial accelerometer-based step counter versus manual recording techniques in which we compared five different step counter placements (wrist and ankle on the non-affected and affected side and waist) in stroke patients. The step counter was tested in a controlled laboratory setting using widely accepted clinical tests that evaluate different activity domains that, according to the International Classification of Functioning (ICF), are predictive of patients’ functional capabilities in the community. A handheld counter was used to count the steps during each clinical test. Manual step counting is a recognized method, and it is considered to be the criterion standard for assessing accuracy [57]. 

In healthy subjects, an excellent correlation was observed when comparing measurements obtained with the step counter and number of steps measured manually in the 50 m WT and in the 6 min WT. A low correlation was observed in 10 m WT and in the TUG test, probably because of the low number of steps performed by the subjects. Therefore, better results were achieved in the long-distance tests, where the number of steps was considerably higher, and the risk of errors registered by the step counter in counting steps was lower. Moreover, better correlations were shown when the device was placed on the lower limbs and on the waist, while there was no statistically significant correlation when the step counter was placed on the upper limbs. Even considering the low number of healthy subjects enrolled in this study, it is possible to conclude that the Nakosite 3D Walking accelerometer-based step counter can effectively be used as a step counter with good reliability in healthy subjects when placed on the lower limbs or on the waist and when used for distances of at least 50 m. 

The gait speed of stroke patients enrolled in this study showed a range between 0.13 and 1.35 m/s. The average speed was 0.75 m/s, which is considerably high when compared with data found in the scientific literature about stroke survivors. The high average gait speed is probably the direct consequence of the inclusion criteria used in this study, because all patients enrolled had to be able to perform the TUG test without direct assistance. This inclusion criterion has inevitably biased our population towards high-functioning patients. The results obtained considering the whole group of stroke patients show that the type of step counter used in this study has poor global accuracy as a step counter with a high failure rate. Only moderate reliability was found for lower limb placement of the device in the 6 min WT.

The Bland–Altman plot confirms our findings for both healthy and pathological groups. As expected, we observed low mean differences for healthy groups (high agreement) and large differences for the pathological group (low agreement).

Accelerometers represent the most common device used to measure variations in acceleration. This measure is more sensitive when the accelerations produced by the patient during the swing phase are higher, i.e., in patients with a high gait speed. If the accelerations are low, the accelerometer is not able to detect variations, and it does not work correctly and has poor sensitivity. To test this hypothesis, it was important to classify patients based on their gait speed and observe if there were differences in accelerometer-based step counter performance. In our study, patients who walked at lower speeds (groups V1 and V2) had poor correlation levels in all clinical tests. The presence of patients walking at low speed meant that the failure rate was high, as the MAPE was high. The low-cost accelerometer-based step counter considered (Nakosite 3D Walking) seems to be a reliable way to count steps only in high-functioning post-stroke patients. Group V3, composed of patients who walked at a speed greater than 0.8 m/s, had excellent ICC correlations and acceptable MAPEs, similar to those measured in healthy subjects.

In the literature, few studies exploring the use of accelerometers and step counters in post-stroke patients can be found [27,37,38,55,58,59]. In most of these studies, it has been confirmed that walking speed is a very important parameter when detecting steps using accelerometers and that lower walking speeds create more problems with step detection [28,36,42]. Campos et al. [59] tried to overcome this limitation of the technology by applying a low frequency extension (LFE) in the post-processing phase of data acquired by accelerometers on stroke patients that theoretically could help to detect steps in slow-walking patients; however, the study did not find an increase in step detection using LFE. 

Our results are also confirmed by Clay et al. [58] and Shaffer et al. [58] who demonstrated a cut-off speed of 0.8 m/s for better accuracy using the Fitbit Zip. Recent studies that used high-tech activity counters, including the ActiGraph GTX3+ [59] and the Sensewear armband [27,36], found better accuracy in step detection in slow-walking stroke patients. All of these accelerometers have a higher retail price compared with the device used in this study. We used the Nakosite accelerometer because, at the time of the study, to the best of our knowledge, it was the lowest cost (15 euros) accelerometer-based step counter on the market both in Italy and the US, easily available on the internet market, and ready to use. This could make it easily accessible in LMIC and usable outside of the clinical setting.

Patients walking at higher speeds (Group V3) had excellent reliability when the step counter was positioned on the lower limbs. This could also be related to the less asymmetric gait in this group of patients. However, we did not perform a study analysis of gait quality in our sample group. This finding is confirmed by Klassen et al. [38], who tested accelerometers in stroke patients and found a more reliable measure on the lower limbs, especially for walking speeds between 0.5 and 0.9 m/s. In our study, ICC correlations for the step counter positioned at the waist were worse than those measured in the healthy subject group, probably due to the differences in trunk kinematics during gait [60]. Schaffer et al. found a good accuracy level for walking speeds over 0.35 m/s using a more expensive accelerometer (Fitbit Zip) worn on the waist [58]; however, they did not test lower limb positioning. Our results are similar to those published by Campos et al. [59], in which the best accuracy was obtained by placing the accelerometer on the ankle of the unaffected side of stroke patients. 

Another important finding of this study is the high reliability of the accelerometer-based step counter in the 6 min WT, confirming the results obtained in the healthy control group. It is necessary for there to be a high number of uninterrupted steps to reduce the percentage of errors in step counting by the step counter. This could also explain the poor reliability of the step counter during the TUG test when compared with the other walking tests. We must consider that subjects performed a mean of 9.6 steps (SD ±1.17) during the TUG test, and an error in step counting of just a single step is more than 10% of the total. Another aspect could be related to the possibility of recognizing postural transitions by the sensors during the TUG test. In the literature, it has been proposed that this limitation could be overcome by using wearable barometric pressure sensors [61]. Patients were also stratified based on walking aids used during walking. As expected, patients differed in walking speed, with those in Group A1 (patients who were able to perform the tests without any walking aid) walking faster. It is noteworthy that when the step counter was positioned on the upper limbs in Croup A3 (patients who needed a walker for ambulation), no steps could be recognized.

The accuracy of using the Nakosite 3D wearable system was excellent for individuals with mild gait impairment following stroke and when the device was placed on the less-affected lower limb. 

Based on the clinical tests performed in the study, these stroke patients were probably able to walk outside of the rehabilitation setting and to perform medium to long periods of uninterrupted walking. It has been emphasized in previous studies that stroke patients with mild gait impairments still have problems with mobility in the community [43]. It remains critically important for these individuals to engage in regular physical activity to counteract sedentary behavior. This is of concern, given that they may arguably represent a higher functioning and more active subset of the stroke population. We should keep in mind that community ambulation post-stroke is a complex behavior involving individual, environmental, and contextual factors [62] that make it challenging to measure [63]. Low-cost step counters could be an effective tool for evaluating physical activity in community-dwelling stroke survivors. 

There are several limitations in this study. First, the healthy group had a significantly lower mean age than the post-stroke patients; therefore, a control group of healthy elderly is needed to confirm the present results. The gold standard considered was manual step counting. Although the operator who performed the manual counting had previous training and was the same for all patients, the procedure included inherently unavoidable human-related error. Moreover, the testing of patients was not filmed in order to perform a visual analysis and differentiate between gait patterns. The performance of a visual analysis in future studies regarding stroke patients and step counters could be important both to improve the accuracy of human manual counting and to better understand how the quality of gait influences the accuracy of step detection [26]. Because of the commercial nature of the device considered, we had no control over the algorithm of the interpretation of raw data. We considered the default algorithm included in the accelerometer-based step counter, because it is the most easily available to utilize on patients, and it is also paradigmatic for commercially available accelerometer-based low-cost step counters that can be found on the market. 

All clinical tests were performed in a clinical setting and not in an ecological setting. However, this limitation is common to most studies of this type performed [38,55,58]. Furthermore, considering a possible future use for daily monitoring of activity, daily adherence was not tested. Adherence could be an issue, as it has been seen that the mental effort needed to remember to wear it every day may not be tolerated by some patients, especially if there is a cognitive impairment, a condition that is often linked to strokes [64]. Strokes can also cause a reduction in dexterity that can limit a patient’s independence in putting on and taking off the device, thus limiting adherence [59]. Furthermore, patients prefer to wear devices that are small and concealed under clothing or integrated into their watch in order to limit social stigma as much as possible [64]. While we adapted the step counter with straps on both ankles and wrists that can probably be concealed during cold periods of the year, if the patients wear less clothing, they could be exposed, thus reducing their acceptability for the patient. 

## 5. Conclusions

When the low-cost accelerometer-based step counter was placed on the less-affected lower limb, good accuracy was shown for step counting in high-functioning stroke patients when walking at a self-selected speed of >0.8 m/s. Step counting could have a practical clinical impact because a change in the number of steps performed per day influences health outcomes. Up until now, the gold standard for inpatient evaluation has been based on clinical outcomes that are able to measure patients’ capacity, but the investigation of real-life performance has not been possible. It remains critically important, for high- and low-functioning stroke patients, to engage in regular physical activity to counteract sedentary behavior and reduce the elevated risk of secondary cardiac complications and recurrent events.

The step counter used in this study could be a valid and reliable option to measure ecological mobility after a stroke in high-functioning patients and could be easily and promptly used in everyday clinical settings due to its low-cost and user-friendly characteristics.

## Figures and Tables

**Table 1:** Sex distribution, age, comfortable walking speeds, and walking aids used by groups V1, V2, and V3 and A1, A2, and A3.

Sex (*n*)				Age (Years)				Comfortable Walking Speed (m/s)				Walking Aids (*n*)		
Subgroup	Males	Females	*n*	Mean	DS	Min	Max	Mean	DS	Min	Max	None	Cane	Walker
**V1**	10	2	12	61.08	20.13	27	84	0.36	0.14	0.13	0.56	1	8	3
**V2**	5	9	14	58.5	14.87	23	81	0.67	0.02	0.64	0.7	8	5	1
**V3**	12	5	17	63.65	9.75	40	77	1.08	3.37	0.86	1.35	14	0	3
**A1**	14	9	23	58.61	15.36	23	77	0.96	0.26	0.42	1.35			
**A2**	9	4	13	59.92	14.26	27	81	0.48	0.18	0.18	0.69			
**A3**	4	3	7	72.43	10.85	55	84	0.63	0.31	0.13	0.97			

**Table 2:** Mean absolute percentage error (MAPE) and intraclass correlation coefficient (ICC) of manually counted steps and steps counted using step counters in different positions in healthy subjects (*n* = 10).

10 m Walking Test—Healthy Subjects					
Measures	Right		Left		
	Ankle	Arm	Ankle	Arm	Waist
**Mean Manual Count** (steps ± SD)	14.60 ± 1.65				
**Mean Step Counter** (steps ± SD)	15.30 ± 1.89	13.30 ± 2.71	14.60 ± 2.17	15.30 ± 6.83	15.00 ± 1.41
**MAPE** (% ± SD)	4.75 ± 4.44	12.78 ± 14.15	6.31 ± 10.04	19.39 ± 30.6	4.52 ± 5.55
**ICC** (95%CI)	0.92 (0.73–0.98)	0.33 (−0.33 to 0.78)	0.46 (−0.19 to 0.83)	0.24 (−0.42 to 0.73)	0.84 (0.50 to 0.96)
**50 m Walking Test—Healthy Subjects**					
**Mean Manual Count** (steps ± SD)	69.30 ± 6.34				
**Mean Step Counter** (steps ± SD)	70.20 ± 6.49	57.60 ± 14.22	70.00 ± 6.24	57.60 ± 12.48	66.50 ± 6.34
**MAPE** (% ± SD)	1.63 ± 1.54	17.49 ± 18.43	1.03 ± 0.97	17.0 ± 15.85	6.32 ±16.51
**ICC** (95%CI)	0.98 (0.93 to 0.99)	0.25 (−0.40 to 0.74)	0.99 (0.98 to 0.99)	0.38 (−0.28 to 0.80)	0.54 (−0.08 to 0.86)
**6 min Walking Test—Healthy Subjects**					
**Mean Manual Count** (steps ± SD)	689.60 ± 52.42				
**Mean Step Counter** (steps ± SD)	695.20 ± 54.08	572.70 ± 99.08	694.00 ± 50.73	534.00 ± 120.42	690.60 ± 51.62
**MAPE** (% ± SD)	1.02 ± 0.83	16.7 ± 14.9	0.66 ± 0.88	22.47 ± 17.81	1.64 ± 3.14
**ICC** (95%CI)	0.99 (0.96 to 0.99)	0.06 (−0.55 to 0.64)	0.99 (0.97 to 0.99)	0.05 (−0.57 to 0.63)	0.92 (0.70 to 0.98)
**Timed Up and Go Test—Healthy Subjects**					
**Mean Manual Count** (steps ± SD)	9.60 ± 1.17				
**Mean Step Counter** (steps ± SD)	10.20 ± 3.9	12.10 ± 1.68	11.50 ± 1.72	12.60 ± 1.90	11.30 ± 0.67
**MAPE** (% ± SD)	26.08 ± 30.59	31.33 ± 16.12	22.56 ± 14.01	33.86 ± 16.49	18.81 ± 11.55
**ICC** (95%CI)	0.21 (−0.45 to 0.72)	–0.03 (−0.62 to 0.58)	0.36 (−0.31 to 0.79)	0.24 (−0.42 to 0.74)	0.51 (−0.13 to 0.85)

**Table 3:** Mean absolute percentage error and intraclass correlation coefficient for manually counted steps and steps counted using step counters in different positions in pathological subjects (*n* = 43).

10 m Walking Test—Pathological Subjects.					
Measures	Healthy		Pathological		
	Ankle	Arm	Ankle	Arm	Waist
**Mean Manual Count** (steps ± SD)	25.38 ± 9.55				
**Mean Step Counter** (steps ± SD)	16.93 ± 9.70	11.32 ± 9.58	18.44 ± 9.17	15.07 ± 8.57	13.23 ± 9.25
**MAPE** (% ± SD)	30.97 ± 38.14	48.56 ± 43.12	29.62 ± 35.37	39.98 ± 37.25	45.08 ± 43.15
**ICC** (95%CI)	−0.19 (−0.46 to 0.11)	−0.41 (−0.63 to 0.13)	−0.20 (−0.47 to 0.10)	−0.50 (−0.34 to 0.26)	−0.42 (−0.64 to 0.14)
**50 m Walking Test—Pathological Subjects**					
**Mean Manual Count** (steps ± SD)	110.91 ± 44.23				
**Mean Step Counter** (steps ± SD)	88.95 ± 48.29	52.44 ± 35.07	90.30 ± 39.34	69.39 ± 43.88	66.83 ± 34.20
**MAPE** (% ± SD)	18.45 ± 33.68	48.96 ± 37.53	18.74 ± 29.34	35.26 ± 38.44	33.30 ± 39.81
**ICC** (95%CI)	0.37 (0.08 to 0.60)	−0.32 (−0.57 to 0.02)	0.06 (−0.24 to 0.36)	0.07 (−0.24 to 0.36)	−0.40 (−0.61 to 0.10)
**6 min Walking Test—Pathological Subjects**					
**Mean Manual Count** (steps ± SD)	536.76 ± 113.29				
**Mean Step Counter** (steps ± SD)	485.76 ± 213.60	309.36 ± 226.62	497.24 ± 218.63	383.61 ± 229.09	418.88 ± 239.06
**MAPE** (% ± SD)	17.13 ± 31.72	48.31 ± 38.51	20.61 ± 29.65	36.60 ± 37.88	31.14 ± 39.22
**ICC** (95%CI)	0.69 (0.50 to 0.82)	0.45 (0.17 to 0.66)	0.70 (0.51 to 0.83)	0.45 (0.17 to 0.66)	0.57 (0.33 to 0.74)
**Timed Up and Go Test—Pathological Subjects**					
**Mean Manual Count** (steps ± SD)	20.38 ± 11.24				
**Mean Step Counter** (steps ± SD)	12.44 ± 9.85	6.93 ± 8.37	12.58 ± 9.42	10.61 ± 9.79	7.02 ± 7.01
**MAPE** (% ± SD)	45.40 ± 41.04	67.81 ± 39.95	42.93 ± 37.15	54.53 ± 39.68	63.79 ± 40.13
**ICC** (95%CI)	−0.28 (−0.54 to 0.01)	−0.23 (−0.49 to 0.08)	0.05 (−0.25 to 0.34)	−0.17 (−0.44 to 0.14)	−0.26 (−0.52 to 0.04)

**Table 4:** Mean absolute percentage error and intraclass correlation coefficient of manually counted steps and steps counted using step counters in different positions in group V3.

10 m Walking Test—Group V3					
Measures	Healthy		Pathological		
	Ankle	Arm	Ankle	Arm	Waist
**Mean Manual Count** (steps ± SD)	19.00 ± 3.79				
**Mean Step Counter** (steps ± SD)	18.94 ± 3.51	11.18 ± 7.63	19.59 ± 2.74	13.65 ± 7.08	15.94 ± 5.17
**MAPE** (% ± SD)	10.05 ± 10.00	37.22 ± 43.11	11.96 ± 15.51	31.23 ± 35.66	20.47 ± 33.89
**ICC** (95%CI)	0.92 (0.73 to 0.98)	0.33 (−0.33 to 0.78)	0.46 (−0.19 to 0.83)	0.24 (−0.42 to 0.73)	0.84 (0.50 to 0.96)
**50 m Walking Test—Group V3**					
**Mean Manual Count** (steps ± SD)	83.63 ± 21.59				
**Mean Step Counter** (steps ± SD)	84.44 ± 20.71	54.19 ± 31.45	84.56 ± 23.12	57.06 ± 34.36	74.56 ± 18.90
**MAPE** (% ± SD)	2.52 ± 2.41	32.19 ± 36.29	3.00 ± 3.85	29.34 ± 37.15	10.39 ± 25.58
**ICC** (95%CI)	0.98 (0.93 to 0.99)	0.25 (−0.40 to 0.74)	0.99 (0.98 to 0.99)	0.38 (−0.28 to 0.80)	0.54 (−0.08 to 0.86)
**6 min Walking Test—Group V3**					
**Mean Manual Count** (steps ± SD)	636.35 ±				
**Mean Step Counter** (steps ± SD)	646.24 ±	409.53 ±	642.82 ±	448.82 ±	591.76 ±
**MAPE** (% ± SD)	1.79 ± 29.58	35.63 ± 37.05	3.31 ± 29.77	30.06 ± 35.68	8.71 ± 37.92
**ICC** (95%CI)	0.99 (0.96 to 0.99)	0.06 (−0.55 to 0.64)	0.99 (0.97 to 0.99)	0.05 (−0.57 to 0.63)	0.92 (0.70 to 0.98)
**Timed Up and Go Test—Group V3**					
**Mean Manual Count** (steps ± SD)	14.94 ± 3.16				
**Mean Step Counter** (steps ± SD)	16.88 ± 3.59	9.18 ± 7.02	14.00 ± 6.74	9.53 ± 7.51	9.18 ± 6.96
**MAPE** (% ± SD)	21.46 ± 19.08	45.18 ± 42.67	30.90 ± 28.41	44.28 ± 42.98	43.55 ± 44.09
**ICC** (95%CI)	0.21 (−0.45 to 0.72)	−0.03 (−0.62 to 0.58)	0.36 (−0.31 to 0.79)	0.24 (−0.42 to 0.74)	0.51 (−0.13 to 0.85)
